# Partial-occupancy binders identified by the Pan-Dataset Density Analysis method offer new chemical opportunities and reveal cryptic binding sites

**DOI:** 10.1063/1.4974176

**Published:** 2017-02-28

**Authors:** Nicholas M. Pearce, Anthony R. Bradley, Tobias Krojer, Brian D. Marsden, Charlotte M. Deane, Frank von Delft

**Affiliations:** 1 Structural Genomics Consortium, University of Oxford, Oxford OX3 7DQ, United Kingdom; 2 Department of Statistics, University of Oxford, 24-29 St Giles, Oxford OX1 3LB, United Kingdom; 3 Kennedy Institute of Rheumatology, University of Oxford, Roosevelt Drive, Oxford OX3 7FY, United Kingdom; 4 Diamond Light Source Ltd., Harwell Science and Innovation Campus, Didcot OX11 0QX, United Kingdom; 5 Department of Biochemistry, University of Johannesburg, Auckland Park 2006, South Africa

## Abstract

Crystallographic fragment screening uses low molecular weight compounds to probe the protein surface and although individual protein-fragment interactions are high quality, fragments commonly bind at low occupancy, historically making identification difficult. However, our new Pan-Dataset Density Analysis method readily identifies binders missed by conventional analysis: for fragment screening data of lysine-specific demethylase 4D (KDM4D), the hit rate increased from 0.9% to 10.6%. Previously unidentified fragments reveal multiple binding sites and demonstrate: the versatility of crystallographic fragment screening; that surprisingly large conformational changes are possible in crystals; and that low crystallographic occupancy does not by itself reflect a protein-ligand complex's significance.

## I. INTRODUCTION

Single-compound high-concentration crystallographic fragment screening experiments are now being increasingly applied to detect the binding of small molecules—“fragments”—to proteins.^1–3^ These experiments entail soaking libraries of fragments with molecular weights of 150–250 Da into protein crystals and determine the resulting structures by X-ray crystallography.^4^ The small size of fragments (∼10 heavy atoms) allows relatively small libraries to efficiently cover chemical space.^5^

However, the small size of fragments means that any binding is inherently weak;^6^ crystallographic occupancies of bound fragments are frequently well below 100%, even when single compounds are soaked at high concentrations.^2^ The presence of crystallographic states may also be substantially affected by crystal effects, e.g., where a conformational change is frustrated by crystal packing constraints, or by crystal cryocooling, which may prevent the detection of “cryptic” binding sites.^7^

Partial occupancy makes it difficult to objectively identify and model bound fragments, since the crystallographic density consists of an average over the bound and unbound states: superposed density corresponding to the unbound state of the crystal may completely obscure evidence for the bound state or merely impede interpretation.^8^ If relying on conventional signal-identification and modelling methods, obtaining clear and interpretable density for the state of interest in general requires time-consuming optimization of crystal systems and/or experimental protocols, and even that may not help.^9^

The problems associated with partial occupancy may be overcome by application of the Pan-Dataset Density Analysis (PanDDA) method,^2^ which reveals clear and interpretable evidence for minor states of the crystal. Through a voxel-by-voxel analysis of the electron densities from multiple crystallographic datasets, binding ligands are identified by contrasting bound datasets against unbound datasets; this allows ligands to be identified with statistical confidence, analogously to isomorphous difference maps.^10^

Once an interesting region has been identified in a particular dataset, the observed electron density at the site remains a superposition of the bound and unbound states of the crystal; subtraction of the superposed unbound density from the crystallographic density subsequently reveals clear density for only the bound state, ensuring that it can be modelled comparatively simply.

We obtain an accurate representation of the density for the unbound state of the crystal from the analysis of multiple unbound datasets; subtracting this density, suitably weighted by an appropriate Background Density Correction factor (BDC), removes the crystallographic superposition and creates a partial-difference map called an *event map*. The event map approximates the electron density for the bound state of the crystal and is thus used for modelling of the bound state.

The PanDDA methodology not only identifies the binding of partial-occupancy ligands but also reveals the ligand-associated conformation of the protein, regardless of how large a conformational change is exhibited upon binding. The PanDDA approach may be applied to any collection of crystallographic datasets of the same crystal form, where a series of unbound datasets are available to provide contrast and thereby identify signal in bound datasets.

## II. DATA AND METHODS

Histone Lysine-specific demethylase 4D (KDM4D) is an epigenetic protein involved in oncology, inflammation, and drug metabolism, and whose down-regulation affects cell proliferation in colon cancer cells.^11,12^ Diffraction data were collected and analyzed for 226 KDM4D crystals, each of which contained a different compound from the Zenobia fragment library (www.zenobiafragments.com). Manual inspection of the conventional difference (*mF_o_-DF_c_*) maps after refinement with a reference model resulted in the identification of two binding compounds, which were subsequently deposited in the Protein Data Bank (PDB)^13^ (PDB codes 4D6R and 4D6S).

A full description of the modelling and refinement of this dataset may be found as part of the original PanDDA manuscript;^2^ however, a brief description of the process is as follows. After automated processing using the PanDDA method with the default parameters, ligand-bound conformations in identified datasets were modelled into the event maps and then merged with a model of the unbound state of the crystal, using alternate conformers to label the different states of the crystal. This process creates an ensemble model of the crystal; the alternate states are crucial to stabilize the refinement of the ligand-bound state.^8^ The resulting ensemble models were refined using typical resolution-dependent protocols, constraining the occupancies of the bound and unbound states to sum to unity where appropriate.

For consistency with the original PanDDA manuscript, the protein is herein referred to as JMJD2D.

## III. RESULTS

**FIG. 1.**
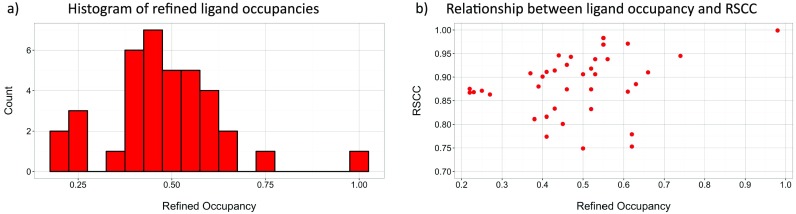
Occupancy and quality of the bound ligand models. (a) Histogram of binding ligand occupancies and (b) the relationship between refined occupancy and the ligand real-space correlation coefficient (RSCC). Although most of the ligands are bound at well below unitary occupancy, the quality of the ligand models remains high, with RSCCs greater than 0.75. Full validation details including the use of additional metrics are listed for each model in supplementary material, Table A2.

Initially, only two ligands were identified to bind to the protein by conventional analysis (datasets x378 and x637), a hit rate of 0.9%. In contrast, analysis with the PanDDA method revealed many partial-occupancy binders that were not visible in the standard *2mF_o_-DF_c_* and *mF_o_-DF_c_* crystallographic maps: 37 binding ligands were detected in 24 datasets, increasing the hit rate by tenfold to 10.6%. Crystallographic data are summarized in Table I; the modelled compounds in each crystal are listed in supplementary material, Table A1. The majority of ligands bind with less than 70% occupancy, but the quality of the ligand models remains high, with real-space correlation coefficients (RSCC) greater than 0.75 (Figure 1). Individual validation plots for each crystallographic ligand, utilizing multiple validation metrics,^8^ may be found in supplementary material, Table A2.

**TABLE I.** Crystallographic summary for the 24 datasets that resulted in models. Multiple crystal structures of this crystal form of JMJD2D have been previously published (e.g., PDBID 4D6R); only a selection of the crystallographic statistics is shown here.

Parameter	Minimum	Lower quartile	Median	Upper quartile	Maximum
Wavelength (Å)	0.920	0.920	0.976	0.976	0.976
Resolution (Å)	1.14	1.27	1.36	1.45	1.97
	29.29	29.34	29.41	29.45	29.51
Spacegroup	P 43 21 2				
Unit cell (Å)	71.12	71.29	71.48	71.53	71.73
	71.12	71.29	71.48	71.53	71.73
	149.74	150.12	150.33	150.57	150.88
Unique reflections	28 265	69663.5	81 352	100018.5	127 202
R-work	0.125	0.128	0.130	0.135	0.162
R-free	0.158	0.162	0.166	0.168	0.219

**FIG. 2.**
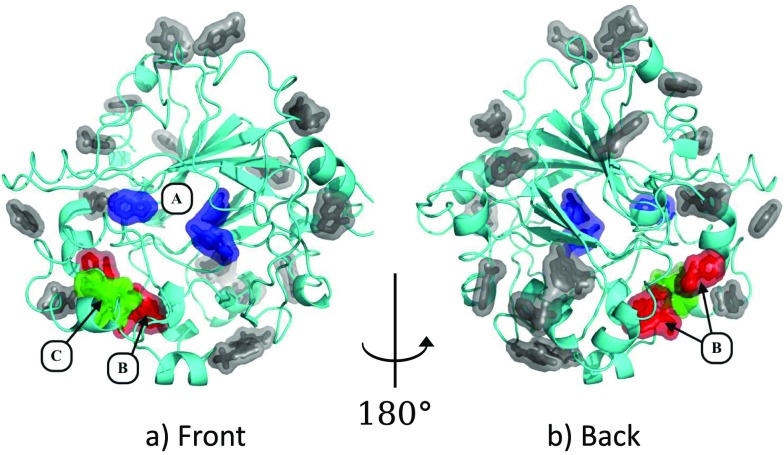
Fragments bind all over the surface of JMJD2D. (a) and (b) Locations of bound fragments. Three sites are highlighted. Site A (blue ligands): three ligands explore the binding site and the peptide binding groove (Section III A). Site B (red ligands): seven ligands bind to a putative allosteric site (Section III B). Site C (green ligands): five ligands consistently stabilize a large conformational change in the terminal helix of the protein (Section III C). The remainder of the binding ligands binds at various points on the protein surface (grey ligands). Images: PyMOL.

Fragments bind at many sites on the surface of the protein (Figure 2), but most binders are singletons, form very few specific interactions with the protein and are likely of little biological relevance (grey ligands; Figure 2). However, there are also several sites that are multiply occupied, the three most significant of which are discussed in this work: the peptide binding region (site A); a putative allosteric pocket on the reverse of the protein (site B); and a cryptic binding site requiring a large conformational change in the terminal alpha helix of the protein (site C).

### A. Binding in and around the orthosteric binding site

**FIG. 3.**
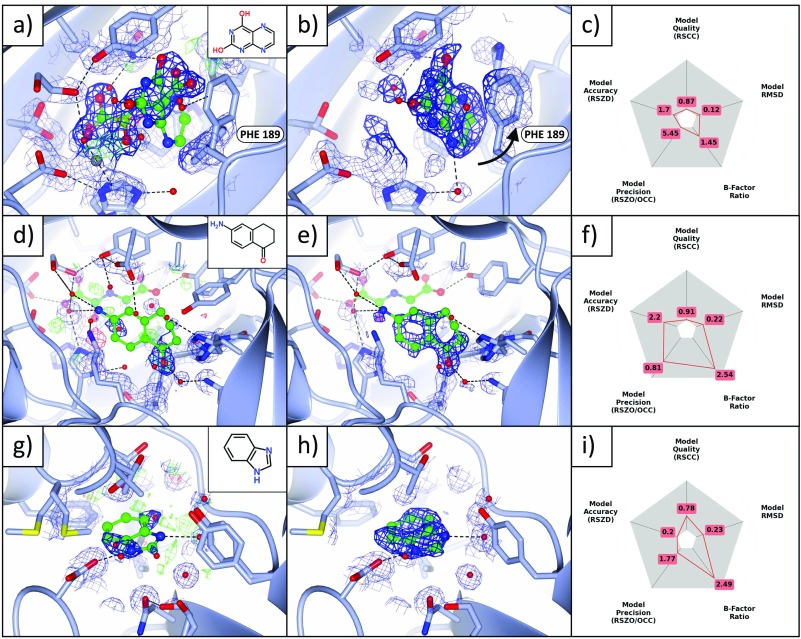
Binding fragments in and around the principal binding site. (a)–(c) Binding to catalytic sites where the metal is absent. (a) The standard *2mF_o_-DF_c_* (blue; 1σ) and *mF_o_-DF_c_* (green/red; ±3σ) maps of dataset x401 (1.48 Å) show no evidence of the ligand (refined occupancy 0.22), even after refinement; only the dominant state, NOG (refined occupancy 78%) bound to the metal (grey sphere, bottom left), is visible. (b) The PanDDA event map (blue, 2σ; BDC = 0.9), however, calculated *before* refinement with the ligand, shows clear evidence for the bound pose (only the bound state of the crystal is shown). The density strength indicates that the metal is not present in this state of the crystal. (c) Validation metrics: the ligand scores well on all metrics. (d)–(i) Two binding fragments in the peptide binding groove. (d)–(f) Dataset x443 (1.43 Å), maps and contour levels as in (a)–(c). (d), (e) The ligand (refined occupancy 0.37) is not visible in the conventional *2mF_o_-DF_c_* map, but is visible in the event map (BDC = 0.92). (f) Instability of the B-factors and occupancy in refinement leads to poor values of RSZO/OCC and the surroundings B-factor ratio. (g)–(i) Dataset x620 (1.25 Å), maps and contour levels as in (a)–(c), except for the event map (BDC = 0.7) which is contoured at 1σ. (g) The ligand (refined occupancy 0.62) is not visible in the *2mF_o_-DF_c_* map, but is visible in (h) the event map, though the density is diffuse: evidence for the ligand is only visible at a lower contour level (1σ). (i) The observed diffuse nature of the density is reflected in the validation metrics, which highlights the high ligand B-factors. The ligand occupies a hydrophobic pocket and makes only one specific interaction with a protein residue and one with a water molecule, both shown in (h), which support the notion that the large B-factors of the ligand may in part indicate real disorder rather than only poor refinement. Images were generated with (a), (b), (d), (e), (g), (h) ccp4mg and (c), (f), (i) matplotlib.

The orthosteric binding site of JMJD2D is occupied by a molecule of N-oxalylglycine (NOG) that, along with the binding site metal, is present at approximately 80%–90% occupancy; this is supported by occupancy refinement of the NOG molecule across the datasets (supplementary material, Figure A1). One fragment (dataset x401) binds in the space vacated by the NOG molecule, but in a perpendicular orientation, and further induces a conformational change in phenylalanine 189 (Figures 3(a)–3(c)). Histidines 192 and 280, which coordinate the binding site metal, become less ordered where the metal and the NOG are absent, but do not change conformation.

**FIG. 4.**
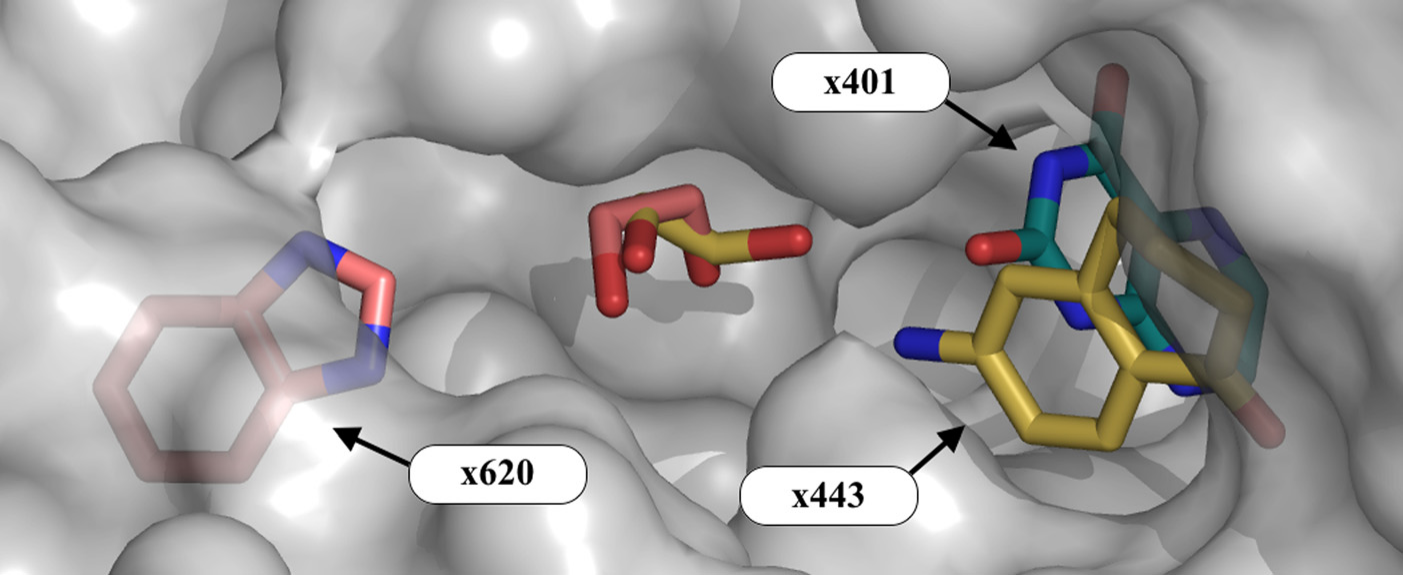
Even the small number of hits sample distinct regions of the orthosteric site. Overview of the binding groove (site A; Figure 2) and the associated ethylene glycol molecules (molecules colored by dataset). The ligand of dataset x401 samples the deep binding pocket, whilst the ligands in x443 and x620 sample sites in the peptide binding groove. The surface of the protein is shown in semi-transparent gray. Images: PyMOL.

Two further fragments (datasets x443 and x620) also bind along the peptide binding groove of JMJD2D (Figures 3(d)–3(f), 3(g)–3(i)); the binding events are not at all evident in the original crystallographic maps but are made clear in the PanDDA maps. Both fragments interact with the protein chiefly *via* hydrophobic interactions, but also make several hydrogen bonds to surrounding water molecules and protein residues. All three compounds sample different parts of the binding groove, yet bind in close proximity (Figure 4), inviting the possibility of linking the fragments.

### B. Detection of a putative allosteric binding site

**FIG. 5.**
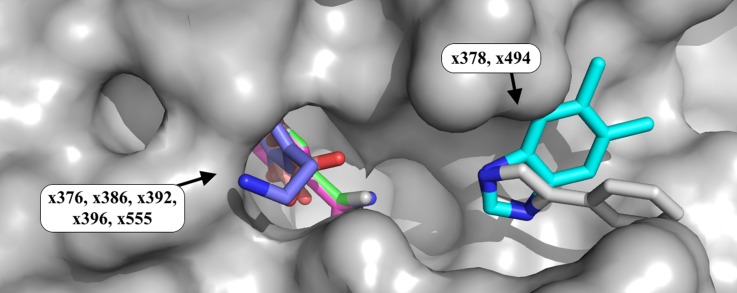
Weakly bound fragments identify a putative allosteric site, with repeated chemotypes. Fragment binding is observed at a putative allosteric site (site B; Figure 2) located on the reverse of the protein from, but still near to, the catalytic site. Five fragments bind in a small pocket, and two bind in a shallow groove on the protein surface. The binding fragments demonstrate repeated chemotypes, such as the superposed 5-membered imidazole rings in datasets x378 and x494. The binding fragment in x386 does not match the soaked fragment; several fragments were misdispensed, and the density is highly similar to that in dataset x376, which also contained a bound ligand. PyMOL colours of carbon atoms of fragments: x376, green; x378, cyan; x386, light magenta; x392, yellow; x396, salmon; x494, grey; x555, slate blue. All other atoms are colored by element. Images: PyMOL.

Seven ligands were observed to bind at a previously unidentified putative allosteric site that is spatially close to the orthosteric site, but accessible from the reverse face of the protein (site B, Figure 2). The binding site consists of a small pocket and a shallow groove on the surface, binding five and two fragments, respectively (Figure 5). The two fragments that bind in the shallow groove (x378 and x494) both contain an imidazole ring, which is present at the same site in both datasets: the conservation of the nitrogen atom positions in the ring indicates their importance to the binding of the two compounds.

**FIG. 6.**
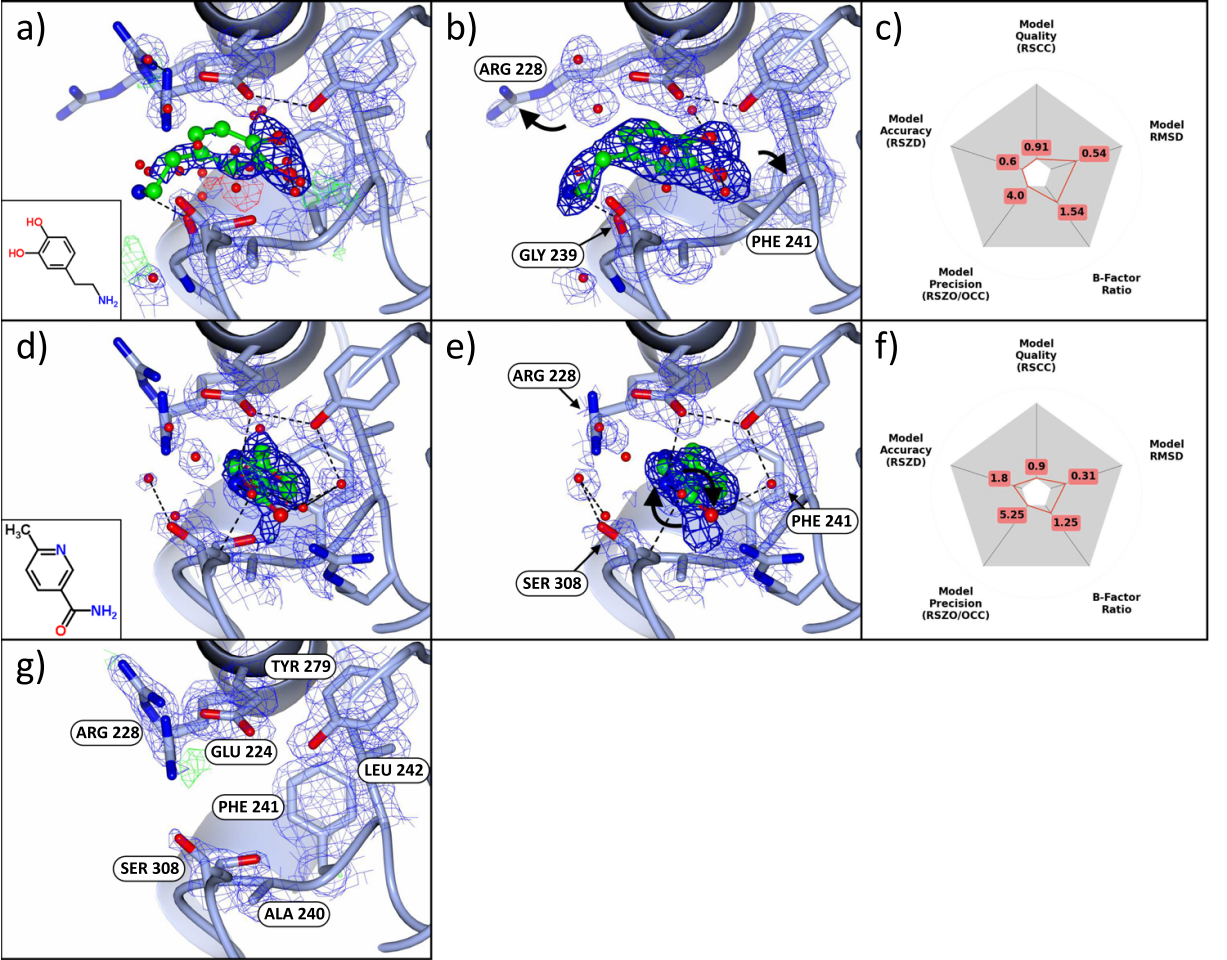
Bound fragments identify putative allosteric sites of the protein. (a)–(c) Dataset x555 (1.60 Å), maps and contour levels as in (Figures 3(a)–3(c)). Once more, the standard *2mF_o_-DF_c_* maps provide unconvincing evidence for the bound state and show a superposition of the surrounding sidechain conformations. The event map (BDC = 0.72) shows clear density for the bound ligand and the displacement of ARG 228 and PHE 241 (indicated with arrows) from the ground-state conformation shown in (g). (d)–(f) Dataset x376 (1.45 Å), maps and contour levels as in Figures 3(a)–3(c). (e) The ligand density is clear in the event map (BDC = 0.78), and unlike (a)–(c), ARG 228 and PHE 241 remain in (a subset of) the ground-state conformations. The ligand restraints enforce the planarity of the ligand in refinement, even though the density clearly indicates a non-planar conformation; the terminal ligand atoms require re-modelling (indicated with arrows) and re-refinement. (g) Dataset x401 (1.48 Å) shows the ground-state conformation of the binding site, for comparison; maps and contours are as in Figure 3(a). Images: ccp4mg.

Two examples of fragments bound in the small pocket are shown for datasets x555 and x376 (Figures 6(a)–6(f)), along with the unbound conformation of the binding site (Figure 6(g)). These binding fragments perturb the sidechains of residues in and around the binding site, sampling conformations of arginine 228 and phenylalanine 241 not visible in the unbound dataset (Figures 6(b) and 6(e)). Other residues are conserved upon binding, except for serine 308, whose conformation is sub-selected from the two conformations observed in the unbound conformation.

The ability of the site to bind multiple ligands displaying similar chemotypes and the short distance to the orthosteric site together indicate that the site may offer an opportunity to allosterically regulate the functionality of the protein. Additionally, the site hosts two adjacent clusters of fragments, suggesting fragment linking as a possible approach for elaborating the bound molecules.

### C. Large binding-stabilized conformational changes

**FIG. 7.**
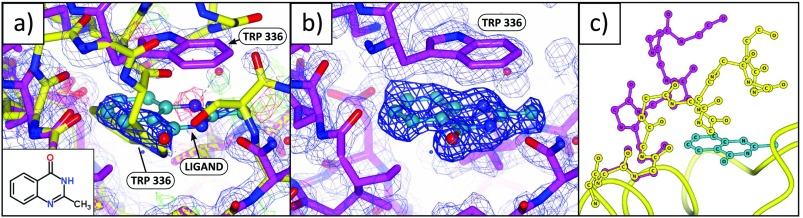
Low-occupancy binders stabilize large structural movements which are only resolved through the use of PanDDA maps. (a), (b) Dataset x402 (1.45 Å), maps and contour levels as in Figures 3(a) and 3(b). (a) Standard crystallographic maps show a superposition of two highly dissimilar states (as modelled), which makes the resulting maps uninterpretable. (b) The event map (BDC = 0.83) shows only the ligand-bound conformation, and enabled modelling of the ligand-bound conformation. (c) The unbound (yellow) and bound (magenta) conformations. Images: ccp4mg.

Several fragments stabilize a large conformational change in the terminal helix of JMJD2D (Site C, Figure 2). All binding events are partial occupancy (refined occupancies 0.39–0.66) and can only be reliably identified by and modelled into PanDDA event maps (Figure 7). Structures were refined as an ensemble of the bound and unbound states except for dataset x395, which is modelled only in the bound conformation; this is due to instabilities in the B-factor and occupancy refinement of the ensemble model, which resulted in significant amounts of residual difference density and inflated model B-factors. Even so, the ligand-bound conformation for dataset x395 is clear in the event maps and does not move under refinement when refined as the sole conformation (refined occupancy 0.66). Furthermore, the compound in x395 differs from the ligand in dataset x393 only by one methyl group and adopts the same binding pose, increasing confidence that the model of the ligand is largely correct.

**FIG. 8.**
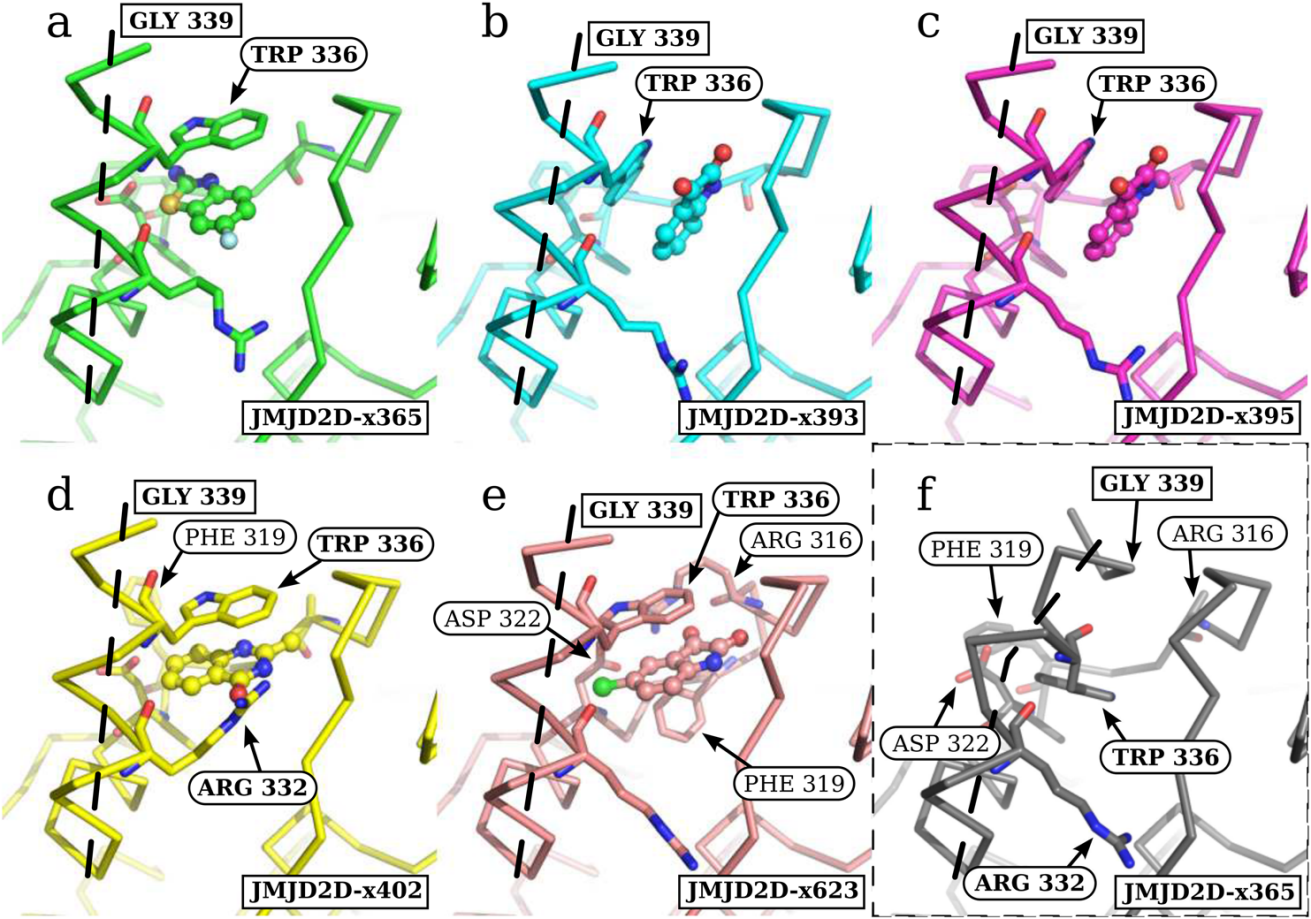
The ligands consistently move the C-terminal helix, but two distinct structural sub-states are observed. (a)–(e) Open fragment-stabilized conformations of the C-terminal helix. (f) Unbound conformation. TRP 336 adopts two distinct sub-states, where the rotamer is (a), (d), (e) changed and (b), (c) unchanged from the (f) unbound conformation. The path of the terminal helix is marked with a dashed line. Interesting residues are labelled in each structure, though the same set of residues are shown as sticks in each image: ARG316, PHE319, ASP322, ARG332, and TRP336. Residues ARG 316, PHE 319, and ASP 322 are more thoroughly discussed in supplementary material, Figure A2. The end of the modelled helix for the bound states (GLY339) is shown in all images. Images: PyMOL.

The bound conformations of the protein form two distinct subgroups, based on the pose of tryptophan 336 (Figure 8). In datasets x393 and x395 (Figures 8(b) and 8(c)), the rotamer of tryptophan 336 is unchanged from the unbound conformation, and only the backbone conformation of the helix changes. Conversely, in datasets x365, x402, and x623 (Figures 8(a), 8(d), 8(e)), tryptophan 336 adopts a rotamer that forms horizontal pi-stacking interactions.

The sidechain of arginine 316 is disordered in all bound structures other than dataset x623, where residues 316–322 adopt a novel conformation, allowing arginine 316 and a new rotamer of aspartate 322 to form a salt bridge (supplementary material Figure A2(e), Figure 8(e)). Arginine 332 is observed to adopt a new conformation in dataset x402, forming interactions with the exposed oxygen atom of the ligand (Figure 8(d)). Residues up to aspartate 341 were resolved in the unbound conformation, but only residues up to glycine 339 were resolved in the PanDDA event maps for the bound conformation for all structures.

## IV. DISCUSSION

In this work, the analysis of one set of crystallographic fragment screening data of lysine-specific demethylase 4D has led to the discovery of a cryptic binding site, a possible new allosteric site, and a more complete mapping of the orthosteric site that shows an opportunity for fragment linking. The previously unknown sites are highly likely to be allosterically relevant, especially since binding occurs near to known functionally important sites. For fragment linking or chemical extension, any binders that probe the chemical potential of nearby regions add further information to guide exploratory efforts.

Crystallographic fragment screening combined with sensitive data-analysis methods has the power to generate large amounts of structural information and reveal exciting structural events for use in protein studies and medicinal chemistry efforts. The management and interpretation of large amounts of structural information poses its own problems; tools such as WONKA^14^ can be used to condense structural information and aid interpretability.

In this work, we show that low occupancy of bound fragments is not an indication of the low value of the corresponding bound structures, but rather the un-optimized nature of fragment screening and restrictions of the crystalline state. Where binding causes energetically unfavorable conformations that may be frustrated by crystal packing, such as the cryptic site discovered here, any binding must be inherently impeded, resulting in reduced occupancy.

Beyond fragment screening, improved data analysis methods such as PanDDA offer a range of new possibilities for probing protein structures and identifying minor states. Historically, signal detection has required large fractions of the crystal to adopt the state of interest; the PanDDA method makes this unnecessary and avoids time-consuming crystal optimization for observing structural events. Furthermore, we have demonstrated that large conformational changes involving multiple residues are possible without disrupting crystal cohesion, showing that dynamic experiments are possible in the crystalline phase.

## V. DATA AND SOFTWARE AVAILABILITY

All ligand-bound structures have been deposited in the PDB under group deposition tool and XChemExplorer (PDB IDs: 5PH0, 5PH1, 5PH2, 5PH3, 5PH4, 5PH5, 5PH6, 5PH7, 5PH8, 5PH9, 5PHA, 5PHB, 5PHC, 5PHD, 5PHE, 5PHF, 5PHG, 5PHH, 5PHI, 5PHJ, 5PHK, 5PHL, 5PHM, 5PHN). An interactive summary of all the ligand-bound structures is available at https://zenodo.org/record/290220/files/0_index.html, which allows users to easily navigate and download the ligand-bound structures: the appropriate event maps are automatically loaded for each structure, which are crucial for making sense of the models. All crystallographic fragment screening datasets are also available on Zenodo (https://zenodo.org/record/48770), to allow reproduction of the PanDDA analysis.

Several conformer-labelling errors have been identified since the publication of the data on Zenodo—though the atomic positions are correct, the conformers corresponding to the bound ligand may not be assigned to the appropriate conformer ID. For example, for a bound ligand with conformer D, the associated conformation may be labelled A or B. These conformer assignments have been corrected in structures deposited in the PDB.

The PanDDA implementation is currently available in the developer version of CCP4 and will eventually be part of the official release version of CCP4; further information and updates will be made available at https://pandda.bitbucket.io.

## VI. SUPPLEMENTARY MATERIAL

See supplementary material for further details of identified ligands and the associated model validation plots.
